# E‑Textiles through a Combination of Laser-Induced Forward Transfer and Electroless Copper Deposition

**DOI:** 10.1021/acsami.5c12675

**Published:** 2025-08-15

**Authors:** Matthias Domke, Justus Landsiedel, Sandra Stroj, Stephan Kasemann, Norbert Lerchster, Margit Lenninger, Ulrich Klapper, Dietmar Holztrattner, Silke Wohnsdorf, Thomas Bechtold, Tung Pham, Noemí Aguiló-Aguayo

**Affiliations:** ‡ 27269 Fachhochschule Vorarlberg, CAMPUS V, Hoechsterstrasse 1, Dornbirn 6850, Austria; † Research Institute for Textile Chemistry and Textile Physics, 27255 University of Innsbruck, Hoechsterstr. 73, Dornbirn 6850, Austria; § Institut für Umwelt und Lebensmittelsicherheit (Umweltinstitut), Montforstrasse 4, Bregenz 6900, Austria; # Adaptive Regelsysteme (ADRESYS) GmbH, Oberndorferstrasse 5, Salzburg 5020, Austria

**Keywords:** conductive textiles, wearables, laser processing, electroless deposition, high-resolution tracks

## Abstract

Electronic textiles (e-textiles) offer promising capabilities in communication, energy storage, safety, comfort, and sensing. A key requirement for e-textiles is the development of conductive patterns with high design flexibility, high electrical conductivity, and strong adhesion to the textile substrates. In this study, a simple approach combining laser-induced forward transfer (LIFT) and electroless copper (Cu) deposition to create high-resolution, highly conductive patterns on textiles is presented. LIFT is used to deposit microsized silver (Ag) seeds onto textiles, offering significant design flexibility for conductive patterns due to the precise control provided by the automated laser system. The silver seeds act as catalysts for subsequent electroless Cu deposition, leading to localized continuous copper tracks with high conductivity. An appropriate spatial distribution of the Ag particles is essential for achieving uniform copper deposition. This was attained through an appropriate design of the donor for the LIFT process. To enhance the adhesion of the Cu coating to the textile, a siloxane-based intermediate layer was introduced between the textile and silver seeds, which significantly improved the adhesion of the copper deposits. As a proof of concept, the methodology was employed for the preparation of inductive antennas on textiles. The combination of LIFT and electroless deposition demonstrates an effective approach for the production of e-textiles.

## Graphical abstract

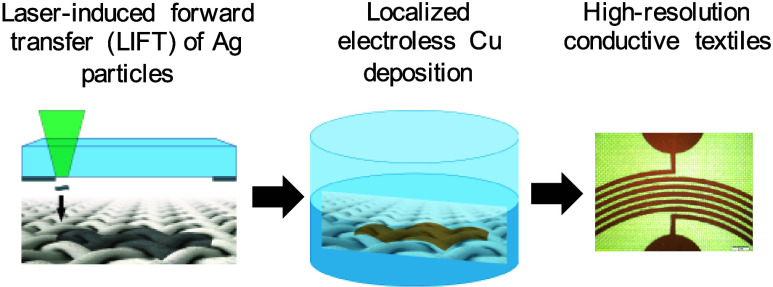

## Introduction

Electronic textiles (e-textiles) offer promising capabilities in a wide range of applications, including wireless communication, efficient energy harvesting and storage, enhanced safety, and advanced sensing capabilities for health monitoring and protective equipment.^1,2^ For these purposes, integrating electrical conductivity into textiles is essential for realizing these capabilities.

There are two primary approaches to achieving this integration. The first approach involves embedding conductive yarns or metallic wires using traditional textile technologies like weaving, knitting, or embroidery.^3–5^ The second approach involves depositing conductive coatings onto flexible substrates using various methods, including spin-coating,^6,7^ screen printing,^8^ inkjet printing,^9^ physical vapor deposition (PVD),^10^ and electroless deposition.^11,12^ While these techniques can create localized conductive patterns, they often require the use of masks (e.g., lithographic processes) and/or post-treatments such as etching or sintering.

The use of mask restricts the resolution of the patterns, and additional processing steps may be required, such as epoxy-like planarization, to enhance the resolution and functionality of the conductive patterns.^13^ Epoxy-like planarization refers to the application of a smooth, leveling layer of material (such as epoxy) to fill surface irregularities and create a more uniform, flat surface. This process, however, reduces the fabric’s natural softness and stretchability by filling the voids and limiting the movement of the yarns.

Some techniques, such as printing, require sintering temperatures above 200 °C to achieve percolation and ensure particle continuity, thereby achieving suitable conductivity.^14–16^ This requirement poses a challenge for temperature-sensitive materials like textiles.

New approaches have been proposed to achieve conductive textiles. Lipovka and coauthors proposed the use of laser processing to form graphene coatings on textiles, achieving a low sheet resistance of (87.6 ± 36.2 Ω/sq) and good adhesion properties.^17^ The procedure consists of impregnating the fabric with a graphene oxide solution and then applying laser irradiation to induce the reduction of graphene. Although the laser processing in general allows for the creation of precise patterns, achieving precision in patterns depends on proper removal of the unfixed graphene, and this may be challenging. Wang and coauthors proposed the use of lithography to create metal patterns on textiles with high precision, achieving sub-100 μm resolution.^18^ However, the process involved first metallizing the entire fabric, then applying a photoresist layer, exposing it to UV light through a photomask, and finally etching the metal beneath the nonpatterned areas using an etching solution. This has the potential to create large quantities of metallic waste, making the process inefficient when aiming to create submicrometer patterns.

In this work, we propose a simple procedure to achieve high-resolution localized conductive tracks consisting of depositing silver nanoparticles directly on textiles in desired patterns using a laser-induced forward transfer (LIFT) technique, followed by electroless copper deposition. LIFT uses a pulsed laser beam for the transfer of material from a donor onto an acceptor.^19^ The donor is immobilized on one face of a glass slide that faces the acceptor (i.e., the textile substrate). The laser pulses are directed from the opposite face, transmitted through the slide, and absorbed at the interface. This leads to a confined ablation that causes the detachment of the donor material.^20,21^ One of the main advantages of LIFT is that almost any material can be transferred, including Ag nanoparticle inks.^22,23^ Electroless copper deposition is a well-known industrial process in which a deposition bath containing a copper salt and a reducing agent initiates a redox reaction, causing copper to deposit onto a catalytic surface. The catalytic surface triggers the reduction of the copper ions to metallic copper, and thus deposition occurs only at locations of the catalytic seeds.^24^ Esrom and co-workers demonstrated that LIFT-transferred Pd layers can serve as catalysts for subsequent electroless Cu deposition on a quartz substrate.^25^ However, the electrical conductivity and mechanical properties of the resulting Cu layer were not reported. In this work, we demonstrate how LIFT can be employed to create localized catalytic surfaces by transferring Ag particles on specific areas of the fabrics, which in turn are used to create high-resolution, conductive Cu tracks for the production of e-textiles. Additionally, we show how using an intermediate siloxane coating significantly enhances the adhesion properties of the Cu-coated tracks on textiles.

## Materials and Methods

### Materials and Chemicals

Investigations were performed on plain-woven polyamide 6,6 (PA6,6) fabrics with a basis weight of 80 g/m^2^ and a yarn count of 110 dtex.

Sol–gel siloxane precursors 3-triethoxysilylpropyl succinic anhydride (TEPSA, Geniosil GF20) and tetraethoxysilane (TEOS, Silikat TES 28) were obtained from Wacker Chemie AG (Burghausen, Germany). Copper sulfate pentahydrate (CuSO_4_·5H_2_O, ≥99.5%), sodium hydroxide (NaOH, ≥98%), potassium hydrogen tartrate (C_4_H_5_KO_6_, ≥99.5%), and formaldehyde solution (≥37 wt %) were sourced from Carl ROTH GmbH & Co. KG (Karlsruhe, Germany). Sodium carbonate (Na_2_CO_3_, ≥99.5%) and nitric acid (HNO_3_, 65 wt %) were acquired from Merck KGaA (Darmstadt, Germany). All chemicals were utilized as received without further purification.

### Procedure for LIFT and Electroless Cu Deposition

For the transfer of Ag particles using the LIFT process, three types of glass donors were evaluated: a single-layer donor with a 20 nm Ag coating on glass (“type 1”), a two-layer donor with a 20 nm Ag and 10 nm SiO_2_ coating on glass (“type 2”), and a three-layer donor with 5 nm Cr, 15 nm Ag, and 10 nm SiO ***_2_*** coatings on glass (“type 3”). Two laser fluences were investigated for each donor. Standard microscopy glass slides were used as the substrates. The donor substrates were prepared by physical vapor deposition (PVD) by using the thermal evaporation technique. A vacuum coating system (BALZERS BAK 550, Balzers, Liechtenstein) with a four-crucible electron beam evaporation source (BALZERS EHV 110, Balzers, Liechtenstein) was used to deposit multiple layers on the glass donors. The different layers were deposited sequentially in a single process run without interruption of vacuum to prevent oxidation of the metallic layers. Film thickness was monitored in situ throughout the coating process by using a quartz crystal oscillator deposition controller (INFICON XTC/2, Bad Ragaz, Switzerland). The LIFT conditions are summarized in Table 1. The peak radius (ω_0_) was found to be 10 μm using the method described by Liu.^26^ The laser power (*P*) was measured behind the focusing optics by using a power meter. The peak fluence was calculated as *F*_0_ = 2*P*/(*f*_rep_ω_0_^2^π), where *f*_rep_ is the pulse frequency of the laser system.

**Table 1:** LIFT Conditions for Investigating the Resolution of Cu Tracks

Condition	Peak Flluence [J/cm^2^]	Donor Substrate
1a	0.15	Type 1: a soda-lime glass substrate coated with a 20 nm Ag layer deposited by thermal evaporation, used as the transfer material
1b	0.30	
2a	0.15	Type 2: a soda-lime glass substrate sequentially coated by thermal evaporation with a 20 nm Ag transfer layer and an additional 10 nm SiO_2_ barrier layer
2b	0.20	
3a	0.09	Type 3: a soda-lime glass substrate sequentially coated by thermal evaporation with a 5 nm Cr adhesion layer, a 20 nm Ag transfer layer, and a 10 nm SiO_2_ barrier layer (topmost)
3b	0.15	

The LIFT experiments were conducted by using an industrial-grade laser processing system (microSTRUCT vario, 3D-micromac, Chemnitz, Germany) with an ultrafast laser source (Spirit HE, Spectra-Physics, Rankweil, Austria). The laser source provides a maximum power of 30 W and emits pulses with a duration of 380 fs. It was operated at a wavelength of 520 nm and a frequency of 100 kHz. The beam was focused to a spot radius of approximately 10 μm, and the peak fluence was set to 1 J/cm^2^. The scan speed was 2 m/s, resulting in a pulse spacing of 20 μm. The line spacing was equal to the pulse spacing. The donors and the fabric receiver were in direct contact during the process with an estimated effective spacing of 10–20 μm between the fabric receiver and the donor substrate. The donor substrates refer to the substrates onto which the Ag transfer layer is deposited, while the receiver substrates are those onto which the Ag particles are transferred. The pattern used in these experiments is presented in Figure 1.

**Figure 1:**
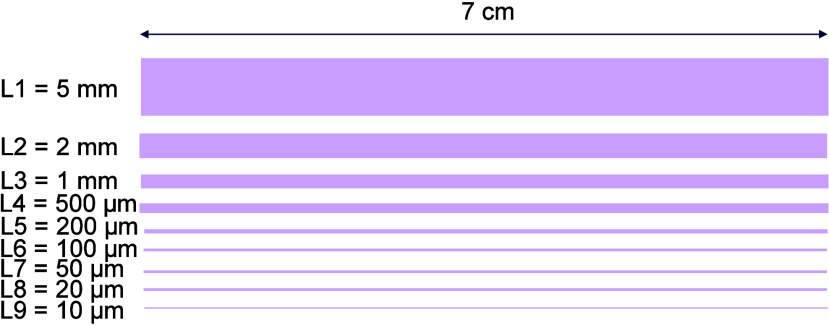
LIFT pattern used for the evaluation of the resolution of the Cu tracks on textiles.

The electroless copper deposition that followed the LIFT process was performed typically for 90 min by immersing samples in a 45 mL electrolyte bath placed in an ultrasonic water bath at 25 °C. The electrolyte bath consisted of 7g/L CuSO_4_·5H_2_O, 12g/L C_4_H_5_KO_6_, 2 g/L Na_2_CO_3_, with pH 12.74 adjusted with NaOH, and 26 mL/L formaldehyde solution.

### Morphological Characterization

The morphological characterization was performed with a field-emission scanning electron microscope (FE-SEM) JSM 7200F (JEOL, Akishima, Japan), a tabletop SEM TM4000 Plus (HITACHI, Tokyo, Japan) equipped with a Bruker Quantax 70 for energy-dispersive X-ray spectroscopy (EDX), a confocal laser scanning microscope (LSM) VKX100 (KEYENCE, Tokyo, Japan), and an Axiotech 100HD-3D microscope (ZEISS, Jena, Germany). For the line-edge roughness analysis, the Analyze Stripe macro in ImageJ software was used.^27^

### Silver and Copper Content Determination

The silver and copper contents were determined using inductively coupled plasma–mass spectrometry (ICP-MS) with an Agilent 7800 instrument (Agilent Technologies, Inc., Santa Clara, United States), following ISO 17294-1. The metal ions were extracted from multiple 1 cm^2^ pieces of the Cu-coated fabrics in 25 mL of a 15 wt % HNO_3_ solution at a temperature of 80 °C for 2 h. For the ICP-MS analysis, the samples were diluted as required with 5 wt % HNO_3_.

### Electrical Characterization

The sheet resistance of the Cu-coated fabrics was determined by using linear four-point probe conductivity measurements. The potential was monitored on a potentiostat (VPS, Biologics, Seyssinet-Pariset, France) as the current was varied. The electrical resistivity (ρ) was calculated using the formula ρ = *GV*/*I*, where *G* is the geometric correction factor and *V*/*I* are the potential/current values, as described in the literature.^28,29^ An illustration of the method and the geometric considerations is included in the Supporting Information, Figure S1a. Two different probes were employed, depending on the track width. For tracks with line widths larger than 500 μm, a probe with 7 mm spacing was used. For tracks with line widths narrower than 500 μm, a smaller probe, with micrometer-scale probe spacing of *s*_1_ = 745 μm, *s*_2_ = 575 μm, and *s*_3_ = 540 μm, was used. The probe diameter was 100 μm. A micrograph of the smaller probe is shown in Figure S1b. For the calculation of the electrical resistivity from the *V*/*I* measured values, an average equidistant spacing of 620 μm was considered. The error bars for the electrical resistivity values account for this estimation.

### Antenna Tests

Radio-frequency identification (RFID) antennas prepared with the methodology described in this work were evaluated for operation in the 13.56 MHz frequency range. The RFID antennas were created following the design shown in Figure 2 and were prepared using the glass donor “type 3”, a peak fluence of 0.2 J/cm^2^, and a scan speed of 1.2 m/s, resulting in a pulse spacing of 12 μm. The distance at which data transfer occurred between the RFID tag (ST25TA02K-P, STMicroelectronics N.V., Plan-les-Ouates, Switzerland) and an NFC transceiver, in our case a smartphone, was analyzed. A rigid reference FR4 antenna was used for a comparison. A picture of the experimental setup is shown in Figure S2 in the Supporting Information. The textile antennas were connected to the NFC chip ST25TA02K-P via metal buttons, as shown in Figure S3 in the Supporting Information. The impedance behavior was measured using a Bode 100 instrument (Omicron Electronics GmbH, Klaus, Austria).

**Figure 2:**
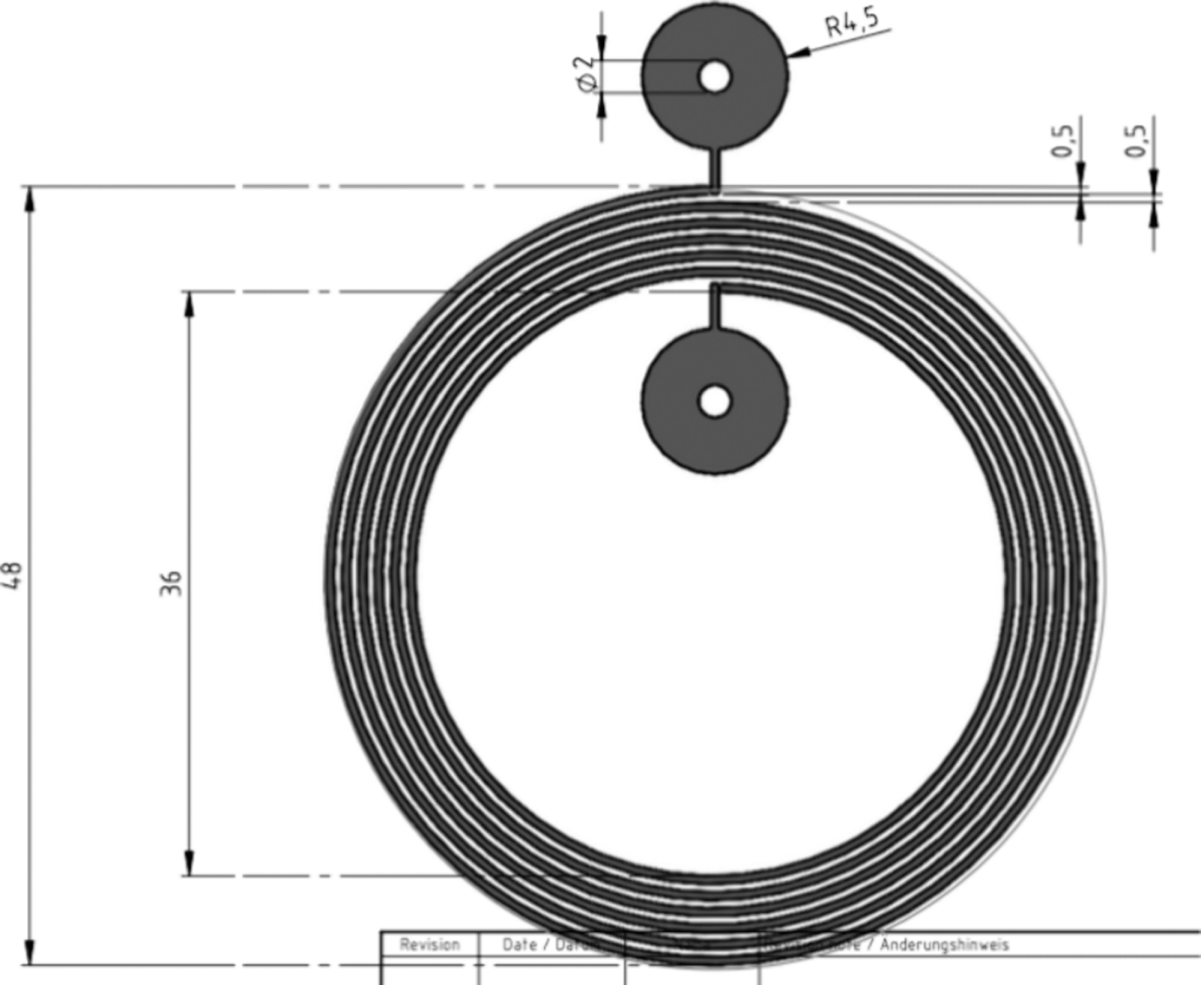
Design of the RFID antenna used for the fabrication of textile antennas (dimensions in mm).

### Adhesion Experiments

Prior to LIFT and electroless copper deposition, a siloxane coating was applied on the fabrics following the procedure described previously.^12^ Two sol–gel siloxane precursors were employed: 3-triethoxysilylpropyl succinic anhydride (TESPSA) and tetraethoxysilane (TEOS). In the first set, 60 mmol of the TESPSA precursor was used; in the second set, 60 mmol of the TEOS precursor was used; and in the third set, a combination of 30 mmol of TESPSA and 30 mmol of TEOS was applied. The rest of the procedure followed exactly what is described previously.^12^

The LIFT process utilized the “type 2” glass donor with a peak fluence set at 0.3 J/cm^2^ to transfer Ag particles onto the textiles. The scan speed was 1.0 m/s, resulting in a pulse spacing of 10 μm. For the preparation of the specimens for T-peel tests, extended electroless copper deposition times—up to the full consumption of the copper ions in the bath solution—were employed to ensure maximum thickness across all samples (a photograph of the bath solution is included in the Supporting Information, Figure S4).

The adhesion of the copper layers was investigated through T-peel tests with a Zwick-Roell Z010 (Ulm, Germany) following the guidelines outlined in ISO 11339:2022. For our experiments, we used specimen lengths of 70 mm, allowing for approximately 50 mm of peeling instead of the standard 100 mm (Figure S5). This adjustment was made to accommodate the specific requirements of our study. The separation rate was 100 mm/min, and the adhesive tape used was polypropylene Tesa 56172 double-sided universal adhesive tape. The tape was applied to the fabrics using a 2 kg roll, which was passed over the adhesive tape two times to ensure proper adhesion. The T-peel test for each sample was conducted immediately afterward.

## Results and Discussion

### Optimization of LIFT Parameters for Ag Seed Transfer

LIFT parameters were investigated to optimize the transfer of Ag particle catalysts onto textiles, thereby enabling selective heterogeneous reduction of Cu^2+^ ions on the Ag seeds during electroless copper deposition. In contrast to studies in the literature that use LIFT to directly print continuous conductive patterns, this work is focused on optimizing LIFT to transfer Ag particle seeds for localized electroless Cu deposition on textiles.

Initial screening experiments (Figure S6, Supporting Information) enabled the identification of suitable LIFT conditions for the present study, including an Ag donor film thickness of 15–20 nm and peak fluences between 0.09 and 0.3 J/cm^2^. These experiments also indicated that the Ag particles were formed during the transfer process.

Figure 3 presents photographs of the Cu-coated fabrics produced under the LIFT conditions outlined in Table 1. LIFT was applied to TEOS-coated PA6,6 woven fabrics. The results indicate that the “type 3” glass donor, which incorporates a Cr layer, enabled the formation of more uniform Cu tracks with enhanced resolution. Lines with line widths up to 50–20 μm could be identified with the naked eyes. Figure 4 compares micrographs of the Cu-coated fabrics obtained with the same laser fluence of 0.15 J/cm^2^ using “type 2” and “type 3” glass donors. When using the “type 3” donor, the Cu layer exhibited improved continuity across all line widths, enabling the formation of tracks as narrow as 20 μm.

**Figure 3:**
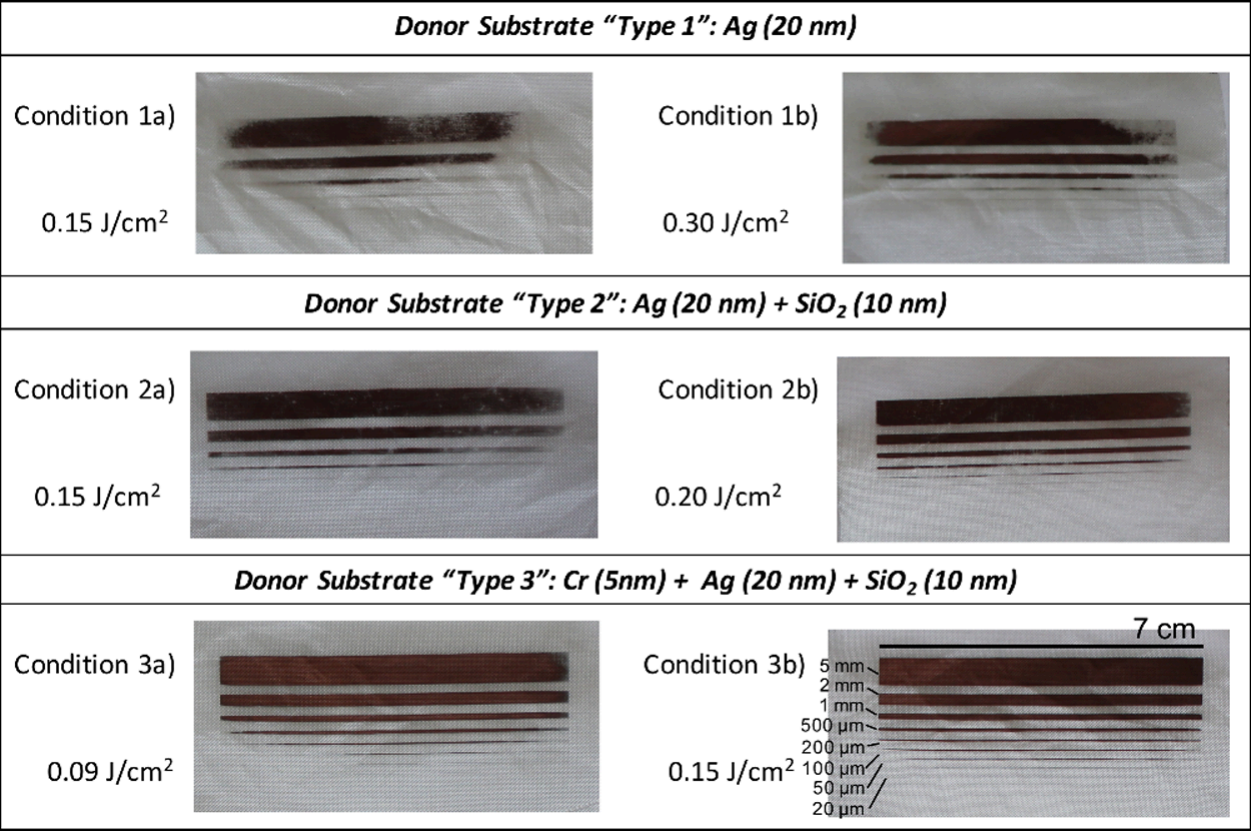
Photographs of the Cu-coated fabrics obtained using different LIFT conditions.

**Figure 4:**
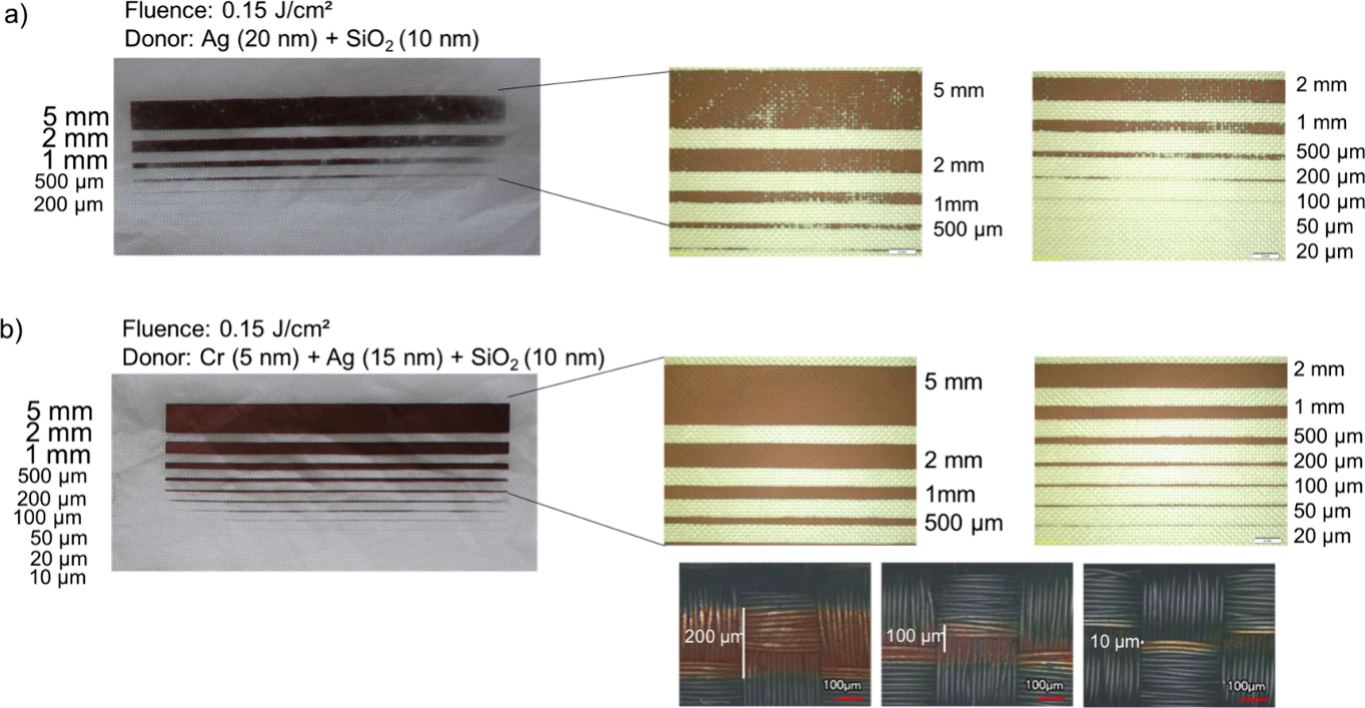
Micrographs of the Cu-coated fabrics using 0.15 J/cm^2^ laser fluence in the LIFT process, but with glass donors (a) “type 2” and (b) “type 3”. The addition of the Cr layer resulted in a higher quality and resolution of Cu tracks on the fabrics.

Table 2 presents the Cu and Ag contents determined by ICP-MS for the different Cu-coated fabrics produced under various LIFT conditions. As expected from the Cu track morphology, fabrics produced with the “type 3” donor exhibited the highest Cu content, ranging from 1.12 to 1.25 mg/cm^2^ (i.e., 140–156 mg/g fabric), which is optimal for achieving proper electrical conductivity in the Cu layers.^12^ Notably, the amount of Ag transferred remained nearly constant across all samples, regardless of the glass donor or fluence applied. This suggests that a specific morphological property of the transferred Ag plays a critical role in enabling the proper formation of the Cu layer. By a “proper Cu layer”, we refer to a continuous Cu coating exhibiting high electrical conductivity. Additionally, the Cr content was below the detection limit of the ICP-MS instrument (<0.2 μg/L), indicating that Cr did not act as a catalyst in the electroless Cu deposition. However, it appears to influence the LIFT transfer of Ag particles from the donor substrate to the receiver, as discussed later. The Ag amount of 7–8 μg/cm^2^, corresponding to approximately 0.1 mg/g of fabric, was the minimum required for the formation of the Cu layer during electroless deposition. This amount of Ag is 10 times lower than the amounts of Ag required in wet chemical processes (>1 mg/g fabric), where the woven fabric is immersed in a silver solution,^12^ which underlines the material-efficient profile of the presented process.

**Table 2:** Ag and Cu Content of the Cu-Coated Fabrics Obtained with ICP-MS

LIFT condition	donor substrate	peak fluence [J/cm^2^]	Ag content [μg/cm^2^]	Cu content [mg/cm^2^]
1a	type 1	0.15	8.6 ± 1.3	0.6 ± 0.2
1b	type 1	0.30	10.6 ± 1.3	0.7 ± 0.3
2a	type 2	0.15	7 ± 3	0.4 ± 0.2
2b	type 2	0.20	7.0 ± 0.9	0.71 ± 0.14
3a	type 3	0.09	6.8 ± 1.3 (Cr < detection limit)	1.12 ± 0.18
3b	type 3	0.15	7.7 ± 0.5 (Cr < detection limit)	1.25 ± 0.3

The morphological characteristics of the transferred Ag particles were investigated using glass slides as receiver substrates, as these allowed better visualization of the Ag particles compared to observations on fabrics. Figure S7 in the Supporting Information shows representative SEM images of the TEOS-coated PA fabrics before and after LIFT. It is reasonable to assume that the spatial distribution on the fabrics is comparable to that observed on glass substrates since the mechanism of particle transfer is independent of the substrate. Nevertheless, the textured surface of the fabrics may locally influence the particle distribution, as evidenced by the characteristics of the Cu layer, which are further discussed in the following section on copper layer morphology.

The electroless Cu deposition on glass slides was unsuccessful due to poor adhesion of the Cu layer, as shown in the Supporting Information (Figure S8a). To address this, TEOS-coated polyamide (PA) films were also tested as flat substrates. Although these films exhibited improved Cu adhesion, delamination of the Cu layer still occurred (Figure S7b). This was attributed to the lower surface area of the films, which limited the mechanical interlocking of the Cu layer. Consequently, glass slides as receivers were used exclusively for characterizing the transferred Ag particles, while investigations of the Cu coating morphology were performed on textile substrates.

The particle size distribution of the transferred Ag particles was primarily determined by the thickness of the Ag film on the donor substrates, as demonstrated in the preliminary studies shown in Figure S6. The donor substrates used in the present study consistently yielded Ag particles with a size distribution identified as suitable for electroless Cu deposition: 100% of the particles with diameters below 1 μm, 80% below 200 nm, and 10% below 100 nm, as illustrated in the histogram highlighted in green in Figure S6a. The Ag particle spatial distribution on the receivers depended on both the peak fluence and the donor type, particularly if the donor included additional layers beyond the Ag transfer layer, such as a SiO_2_ barrier layer or a Cr adhesion layer.

Figure 5 presents micrographs of both single pulses and continuous lines produced under the LIFT conditions of Table 1. The SEM images of the single pulses indicate that the “type 3” donor substrate enabled a more confined transfer of Ag particles, resulting in a narrower spread compared to that of the other donors (Figure 5a). The additional SiO_2_ and Cr layers on the donor substrate improved the adhesion of the Ag coating, reducing the peeling of the irradiated Ag portion on the donor after the vapor expansion occurred. This improvement is evident in the SEM images of the donor substrates and receivers following single pulse irradiation, where the 15 μm spot diameter on the irradiated donor closely matched the diameter of the circular Ag transfer pattern observed on the receivers when using the “type 3” donor, highlighting the efficiency of the transfer process under these conditions. By “transfer efficiency”, we refer to the successful detachment and transfer of Ag particles from the donor to the receiver substrate, resulting in a clean donor crater and a well-defined deposition pattern. The increased transfer efficiency enabled a more controlled transfer of the Ag particles, contributing to a more uniform distribution, typically spreading out in a well-defined circular pattern. This uniformity was also observed during the transfer process involving overlapping pulses (Figure 5b), particularly at higher fluences, where a more consistent deposition of the Ag particles was achieved with the “type 3” donor and no gaps were observed between pulses. Additional SEM images of the glass donors and dark-field microscopy images of the glass receivers after the LIFT process involving overlapping pulses are included in the Supporting Information (Figures S9 and S10). In summary, the LIFT conditions presented in Figure 5 resulted in an equivalent Ag size distribution, as evidenced by the SEM analysis, and Ag yield, as shown by the ICP-MS data. However, they exhibited a different spatial distribution on the receivers, which impacts the electroless Cu deposition. The “type 3” donor with a peak fluence of 0.15 J/cm^2^ provided the most suitable Ag spatial distribution for electroless Cu deposition.

**Figure 5:**
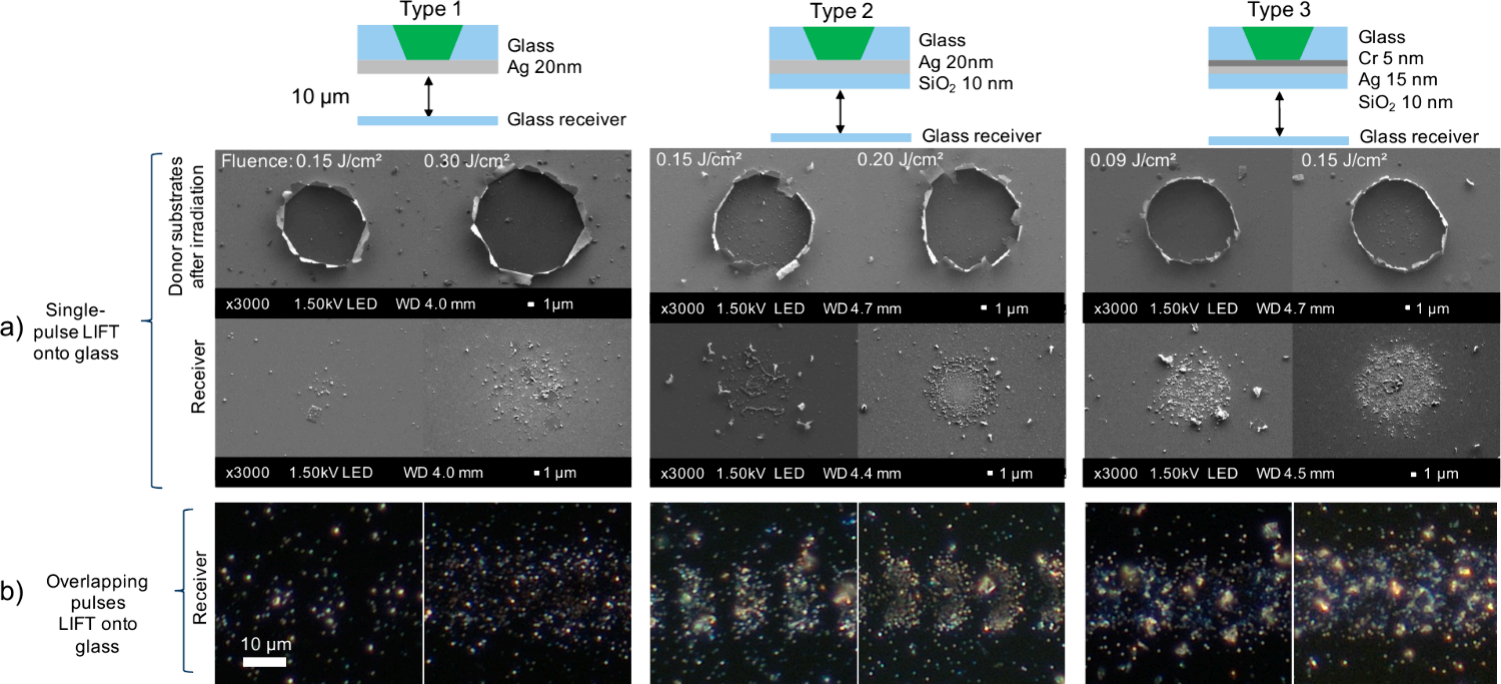
Micrographs of the Ag particles transferred on glass acceptors under the different LIFT conditions, showing results for single pulses (a) and overlapping pulses using a pulse spacing of 10 μm (b). In the schematic, the green shading indicates the beam path of the laser.

### Morphological Characteristics of the Cu Coating under Optimal LIFT Conditions

Figure 6 shows SEM-EDX micrographs of the 100 and 200 μm Cu tracks fabricated under the optimal LIFT conditions mentioned above (condition 3b). Additional SEM and LSM images are available in the Supporting Information (Figure S10). At higher magnifications, the TEOS film and some TEOS particulates can be observed on the non-Cu-coated regions, as highlighted in Figure 6 and in Figure S7. Further details on the siloxane intermediate layer are available in our previous work.^12^

**Figure 6:**
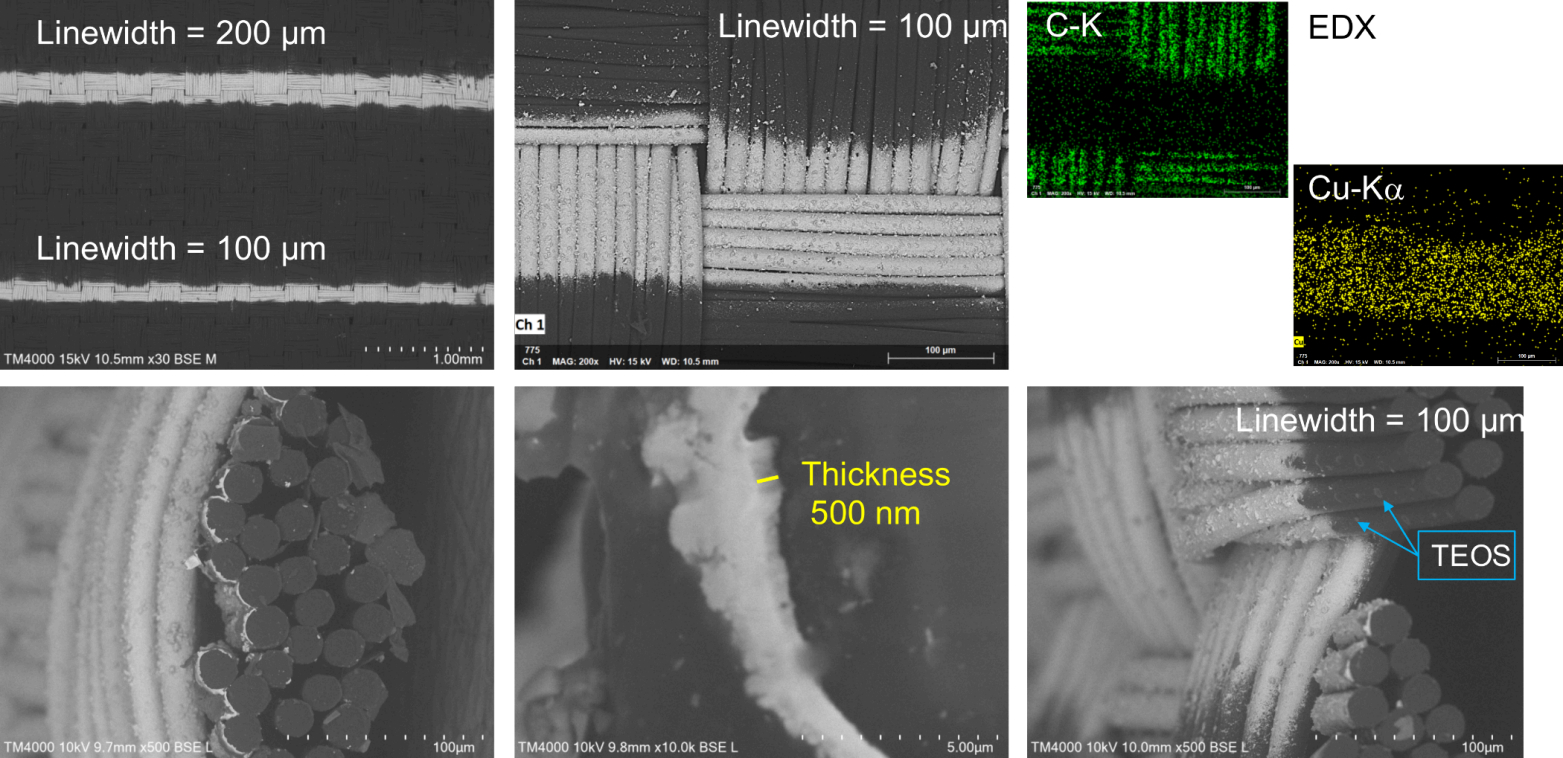
SEM-EDX micrographs of the Cu tracks with line widths of 200 and 100 μm, obtained using the optimal LIFT conditions (condition 3b in Table 2). The thickness of the Cu coating is 500 nm.

SEM analysis reveals that the Cu layer forms a compact and uniform coating with an average thickness of approximately 0.5 μm. Some deviations from a perfectly straight line at the track edges are noticeable. The root-mean-square line-edge roughness (LER_RMS_) was evaluated for different line widths; the corresponding values are summarized in Table 3 and visualized in Figure 7. For line widths up to 200 μm, LER_RMS_ values remain around 20 μm. In contrast, for line widths below 100 μm, LER_RMS_ increases to approximately 50 μm, with higher standard deviations. The relatively high LER_RMS_ is attributed to the inherent textured pattern of the textile substrate. Repeating ridge-like features can be observed at intervals of ∼200 μm along the Cu tracks, coinciding with the intersections of the warp and weft yarns. At line widths below 100 μm, these structural discontinuities increasingly affect the Cu layer’s edge definition, leading to higher LER_RMS_ values. These ridges are not observed when the PA film is used as the substrate. Figure 8 compares a 5 mm line width fabricated on textiles with one on a PA film. Although Cu adhesion to the flat PA film is weaker, due to the reduced surface area, the LER_RMS_ values on the PA film are 10 times lower, 1.3 μm, reinforcing the conclusions that the LER_RMS_ is predominantly influenced by the substrates’s surface characteristics. Future investigations will focus on strategies to reduce LER by minimizing substrate-induced roughness effects.

**Figure 7:**
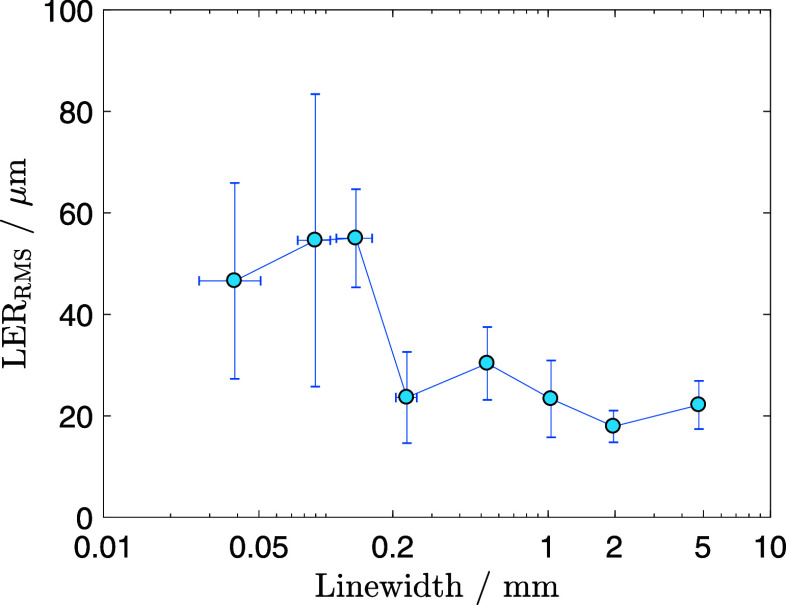
LER_RMS_ values as a function of the Cu line width.

**Table 3:** Line Width and Corresponding LER_RM_ Values Represented in Figure 7

average line width (mm)	LER_RMS_ (μm)
4.76 ± 0.03	22 ± 5
1.96 ± 0.04	18 ± 3
1.03 ± 0.04	23 ± 8
0.53 ± 0.03	30 ± 7
0.23 ± 0.03	24 ± 9
0.14 ± 0.02	55 ± 10
0.090 ± 0.015	54 ± 29
0.039 ± 0.012	46 ± 19

**Figure 8:**
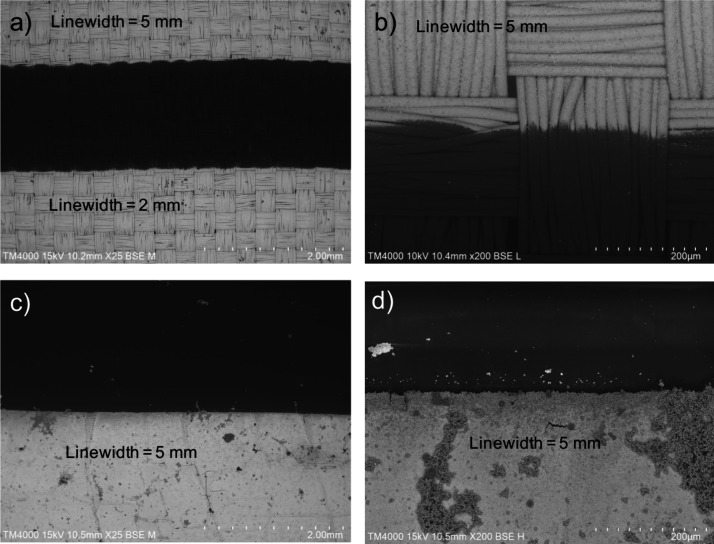
SEM images of the Cu coatings with 5 mm line widths at 25× and 200× magnification, obtained on the fabric (a, b) and on the TEOS-coated PA film (c, d).

### Electrical Properties and Antenna Tests

Figure 9a and Table 4 display the electrical resistivity obtained from the Cu tracks produced under optimal LIFT conditions. The average electrical resistivity for line widths ranging from 5 mm to 200 μm was (17 ± 4) × 10^–8^ Ωm, corresponding to a sheet resistance of 0.34 Ω/sq at 0.5 μm Cu thickness. While structural fabrication down to 20–50 μm was successfully demonstrated, conductivity measurements at this scale remain challenging due to probe size limitations and the discontinuities in the Cu layer caused by the intrinsic porosity of the textile substrate (e.g., at warp and weft crossings). Future work will focus on overcoming these constraints.

**Figure 9:**
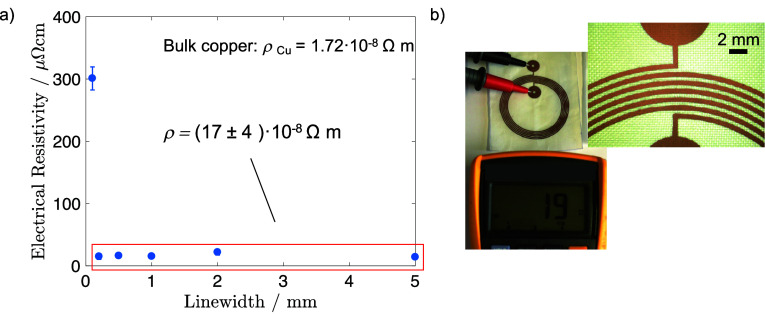
(a) Electrical resistivity of the Cu tracks obtained under optimized LIFT conditions as a function of the Cu line width. The resistivity values were calculated from the sheet resistance (*R*_s_) measured by the four-point probe, using the formula ρ = *R*_s_/thickness. (b) Resistance measurement of 19 Ω for an antenna prototype fabricated with the optimized LIFT conditions. The Cu track had a thickness of 500 μm and a length of approximately 788 mm.

**Table 4:** Data Corresponding to the Electrical Resistivity vs Line Width Plot in Figure 9

line width (mm)	electrical resistivity (Ωm)
5	(14 ± 3) × 10^–8^
2	(21 ± 5) × 10^–8^
1	(15 ± 2) × 10^–8^
0.5	(16 ± 2) × 10^–8^
0.2	(15 ± 5) × 10^–8^
0.1	(302 ± 19) × 10^–8^

The measured resistivity is approximately 10 times higher than that of bulk Cu (1.72 × 10^–8^ Ωm) but comparable to values reported for printed Cu films sintered at 200–350 °C.^14–16^ Such processing temperatures are incompatible with polymeric materials commonly used in textiles, in our case PA66 fabrics, which have a melting point of approximately 220 °C. The measured resistivity is also comparable to that of Cu microcircuits prepared by arc-beam LIFT.^30^ Huang and coauthors reported a value of 14.89 × 10^–8^ Ωm for a 3 times deposition on a glass substrate, although with thicker Cu coatings. The height of the microcircuit from a single deposition was reported to be 0.6 μm, so three depositions yielded a total thickness of approximately 1.8 μm, which exceeds the thickness obtained in our process combining LIFT with electroless Cu deposition.

Figure 9b shows the resistance of antenna prototypes featuring Cu tracks with a line width of 500 μm and a length of approximately 788 mm using a multimeter, which was approximately 19 Ω. Although this value was higher than the resistance of the reference FR4 antenna (Figure S2), only 1 Ω, it aligns with the electrical resistivity values derived from tracks ranging from 1 to 5 mm.

Regarding the performance of the antenna prototypes, communication with the NFC reader was established at distances between 2.5 and 3.5 mm, shorter than that of the rigid FR4 reference antennas, which ranged from 4 to 4.5 mm, as a consequence of the greater resistances exhibited by the textile antennas. It should be noted that the strategies used to connect the NFC chip to the textile antennas via metal buttons (Figure S3), in order to avoid soldering directly onto the textile antenna, further increased the resistance of the prototypes to 60 Ω and in some cases even compromised the contact. The impedance responses of the antennas are shown in the Supporting Information (Figure S12). Although both antennas exhibited a response around the operating frequency at 13.56 MHz, the reference antenna demonstrated better impedance characteristics due to its lower resistance. How to effectively connect the inductive textile antenna to the NFC chip remains a challenge that needs to be addressed.

To demonstrate the potential applicability of the method to other types of fabrics, we also fabricated Cu-coated knitted fabrics (Figure S13). Although the method appeared to be successful and the Cu layer was formed at the desired locations, the resistance was 25 Ω over a 5 cm length for 5 mm tracks, which is lower than that of the woven fabrics. The results obtained with knitted fabrics suggest that further process optimizations may be necessary when different fabric substrates are employed. We believe that increasing the amount of Ag particles transferred could improve the Cu layer formation and thus improve electrical conductivity. However, optimization across various fabric substrates is beyond this study.

### Adhesion Properties of the Copper Coating

From previous investigations, we observed that pretreating polyamide fabrics with an intermediate siloxane coating improved the electrical conductivity and uniformity of the copper layer on polyamide fabrics without altering key fabric properties such as air permeability or flexural rigidity.^12^ In this work, we examined the effect of the siloxane intermediate layer on the adhesion properties of copper coatings on textiles. To ensure a consistent comparison across samples, electroless Cu deposition was performed for extended periods until all of the copper was exhausted from the solution. Extended deposition times resulted in the thickest Cu coatings for all substrates with thicknesses up to 7 μm. SEM micrographs of the cross section of the Cu-coated fabrics without and with a siloxane (TEOS) intermediate layer are shown in Figure 10a,b. Additional SEM-EDX images for Cu-coated fabrics with the different siloxane coatings can be found in the Supporting Information (Figure S14).

**Figure 10:**
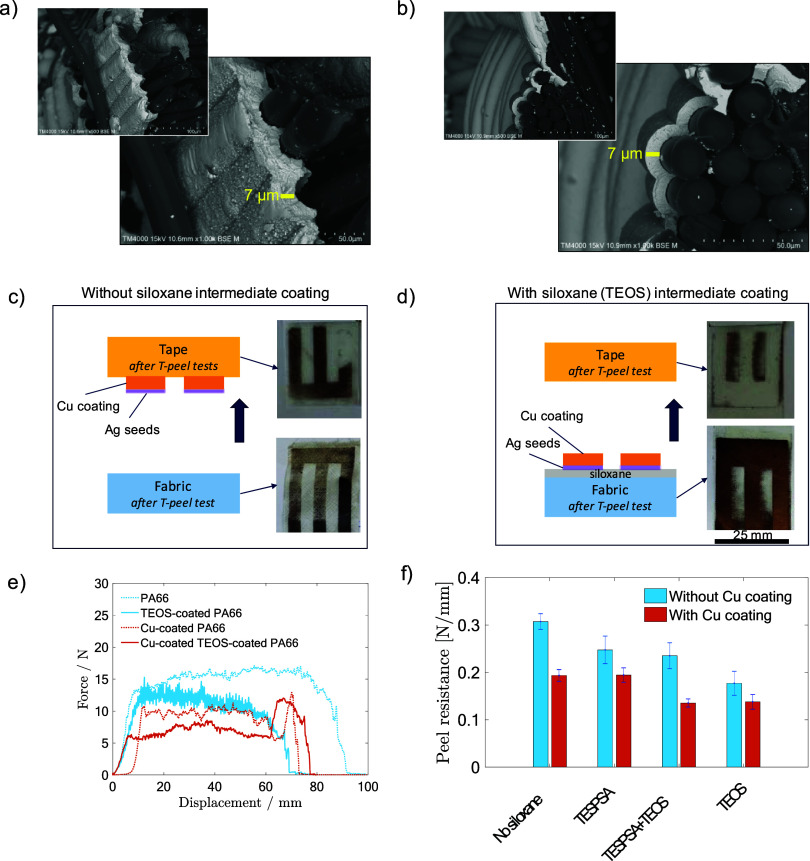
SEM micrographs of the cross section of Cu-coated fabrics: (a) without siloxane and (b) with a TEOS intermediate layer. Photographs of the adhesive tapes and fabrics after T-peel tests: (c) without and (d) with the TEOS intermediate layer. (e) Peel force versus displacement curves from the T-peel tests. (f) Peel resistance (peel force divided by displacement) of the different samples.

The thickness of coatings is one factor influencing their adhesion, as residual stress accumulates with increasing thickness.^31^ Consequently, the thickest coatings present the most critical conditions for assessing adhesion properties. However, prolonged deposition times promoted copper particle migration leading to nonselective deposition beyond the intended areas. This negatively affected the resolution of the conductive tracks and resulted in copper deposition, even in areas without silver catalysts. Interestingly, the presence of copper in areas without silver also allowed for a clearer observation of the improvement in adhesion in regions where both silver and siloxane intermediate layers was present. Without the siloxane, the Cu tracks adhered to the tape, whereas in areas with siloxane, particularly when using TEOS, the tracks remained firmly attached. Figure 10c,d shows photographs of the tapes and fabrics after T-peel tests, both with and without a siloxane coating (TEOS). Additional photographs of the tapes after T-peel tests for the different siloxane coatings are provided in the Supporting Information (Figure S15). The presence of TEOS enhanced the adhesion of the copper coating, as no copper was removed, where the silver catalysts were present. The use of siloxane as an adhesion promoter between polymers and inorganic surfaces, especially in metal coatings, is well-established.^32–34^ Silicon alkoxide pretreatments are known to enhance adhesion and interfacial stability by facilitating the formation of strong covalent bonds. These bonds result from condensation reactions between hydroxyl groups on the treated substrate’s surface and silanols from the hydrolyzed silane. In our case, the adhesion between the metal coating and the siloxane layer is primarily attributed to hydrogen bonds between the siloxane layer and the oxides and hydroxides of the Ag seeds deposited via LIFT.^35,36^ The peel forces required to separate the adhesive tape from the different fabrics (with and without copper coating) are shown in Figure 10e,f and Figure S16 in the Supporting Information. We observed that the presence of TEOS significantly decreased the peel resistance of the copper coatings (as well as for the fabrics without copper coating), while the Cu coating on the LIFT-treated areas remained unaffected and retained its conductivity. The superior enhancement observed with TEOS compared to the TESPSA precursor was attributed to its molecular structure, which features a compact arrangement of four ethoxy groups attached to a central silicon atom, resulting in less steric hindrance than that of the TESPSA precursor (Figure S17a), making TEOS the preferred choice for the intermediate coating. A reaction scheme of the TEOS precursor applied to the polyamide fabrics is shown in Figure S17b in the Supporting Information. The impregnation of PA6,6 fabrics with a prehydrolyzed alkoxysilane solution leads to the formation of thin siloxane films via sol–gel condensation reactions.^37^ During this process, the siloxane networks interact with the polyamide surface through hydrogen bonding, polar interactions, and physical entanglement (Figure S17b).^12,38,39^ These adhesion tests provide a foundation for future investigations, which will include electromechanical cycling tests such as bending and stretching, to evaluate resistance changes under deformation.

## Conclusions

In this study, we present a simple method that combines laser-induced forward transfer (LIFT) and electroless copper (Cu) deposition to create high-resolution, highly conductive patterns on textiles. The process was investigated on woven fabrics and also appears to work on knits, but further optimization is required. To achieve high-resolution Cu tracks, it was essential to transfer Ag particles with a more uniform spatial distribution, typically spreading out in a well-defined circular pattern. This was accomplished through a well-design LIFT glass donor that integrates multiple layers of Cr, Ag, and SiO_2_ to ensure a controlled transfer of the Ag particles. Additionally, the process enabled the creation of highly conductive Cu tracks with an electrical resistivity of (17 ± 4) × 10^–8^ Ωm, corresponding to a sheet resistance of 0.34 Ω/sq at 0.5 μm Cu thickness, using a minimal Ag amount of 7–8 μg/cm^2^, highlighting the material-efficient nature of the process. Textile antenna prototypes, measuring approximately 788 mm in length and line widths of 500 μm, were prepared using this technique, demonstrating its potential. Adhesion tests performed on Cu-coated fabrics with different siloxane intermediate coatings demonstrate improved adhesion when using a TEOS precursor.

## Supplementary Material



## Notes

Scheme of the four-point probe conductivity measurements with geometrical considerations (Figure S1); additional description of the experimental setup for the antenna tests (Figures S2 and S3); photograph of the electroless copper deposition performed on the T-peel test specimens (Figure S4); photograph of the T-peel test and representative specimen (Figure S5); preliminary LIFT studies to select conditions for the present experiments (Figure S6); SEM images of the TEOS-coated polyamide fabric before and after LIFT (Figure S7); micrographs of the Cu-coated glass slide and the TEOS-coated polyamide film (Figure S8); SEM images of the glass donors after continuous laser irradiation (Figure S9); dark-field optimal images of the glass receivers after the LIFT process using overlapping pulses with a pulse spacing of 10 μm (Figure S10); additional SEM and LSM images of the Cu-coated fabrics obtained under optimal LIFT conditions (Figure S11); impedance response of the FR4 rigid reference antenna and the textile-based antenna (Figure S12); Cu-coated knitted fabrics prepared with the present method (Figure S13); cross-sectional SEM-EDX photographs of the specimens for the T-peel tests (Figure S14); pictures of the adhesive tapes after T-peel tests (Figure S15); additional peel force vs displacement curves from the T-peel tests (Figure S16); chemical structures of the TESPSA and TEOS precursor and reaction scheme of the TEOS precursor to the polyamide fabric (Figure S17) (PDF)

## Abbreviations

e-textiles: electronic textiles
LIFT: laser-induced forward transfer
PA6,6: polyamide 6,6
TESPSA: 3-triethoxysilylpropyl succinic anhydride
TEOS: tetraethoxysilane
PVC: physical vapor deposition
FE-SEM: field-emission scanning electron microscopy
SEM: scanning electron microscopy
EDX: energy-dispersive X-ray spectroscopy
LSM: confocal laser scanning microscopy
ICP-MS: inductively coupled plasma–mass spectrometry
RFID: radio-frequency identification
