# Supplementary material for: E‑Textiles through a Combination of Laser-Induced Forward Transfer and Electroless Copper Deposition
Source: ACS Appl Mater Interfaces. 2025 Aug 15;17(34):49038–48. doi: 10.1021/acsami.5c12675 (PMC12400278; doi:10.1021/acsami.5c12675)
Supplement: Supplementary file 1 [file am5c12675_si_001.pdf]

Supporting Information

E-textiles through combination of laser-induced forward transfer and electroless copper deposition

Matthias Domke‡, Justus Landsiedel†, Sandra Stroj‡, Stephan Kasemann‡, Norbert Lerchster§, Margit Lenninger†, Ulrich Klapper#, Dietmar Holztrattner#, Silke Wohnsdorf#, Thomas Bechtold†, Tung Pham† and Noemí Aguiló-Aguayo†*

*Email: noemi.aguilo-aguayo@uibk.ac.at

*orcid.org/0000-0003-0020-0769

AUTHOR ADDRESS

‡Fachhochschule Vorarlberg, CAMPUS V, Hoechsterstrasse 1, Dornbirn 6850, Austria.

†Research Institute for Textile Chemistry and Textile Physics, University of Innsbruck, Hoechsterstr. 73, 6850 Dornbirn, Austria.

§Institut für Umwelt und Lebensmittelsicherheit (Umweltinstitut), Montforstrasse 4, 6900 Bregenz, Austria

#Adaptive Regelsysteme (ADRESYS) GmbH, Oberndorferstrasse 5, 5020 Salzburg, Austria

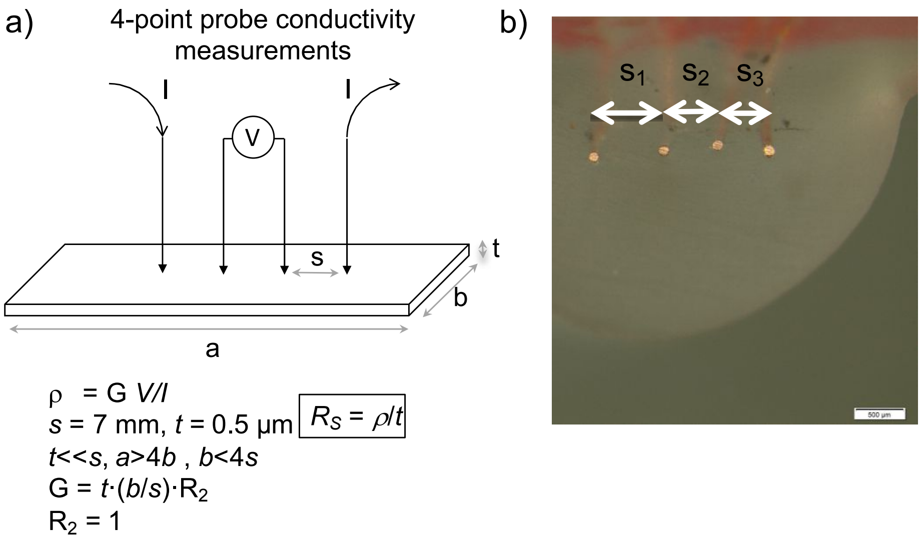

Figure S1. (a) Geometrical considerations for 4-point probe conductivity measurements. Two different probes were used depending on the track width. For tracks with linewidth larger than 500 µm, a probe with 7 mm spacing was used. For tracks with linewidths narrower than 500 µm the probe shown in (b) was used. (b) Micrograph of the four-point probe used for measuring the electrical conductivity of the thinner tracks, with probe spacing of s_1 = 745 µm, s_2 = 575 µm and s_3 = 540 µm. The probe diameter was 100 µm. To simplify the calculation of the resistivity from the *V/I* measured values, an average equidistant spacing of 620 µm was considered. The error bars on the electrical resistivity in Figure 9a account for this estimation.

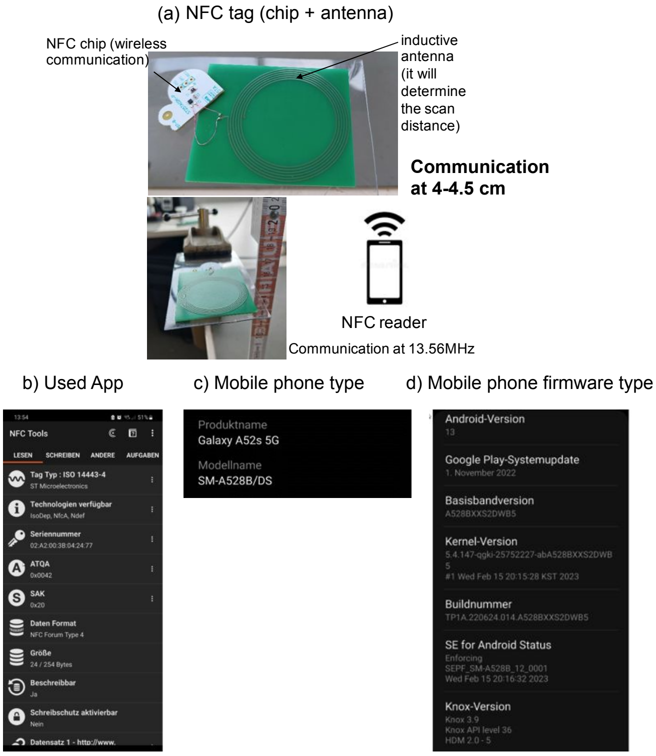

Figure S2. NFC tag (chip + antenna) using reference FR4 as antenna (a) and experimental setup details for antenna testing (b, c, d).

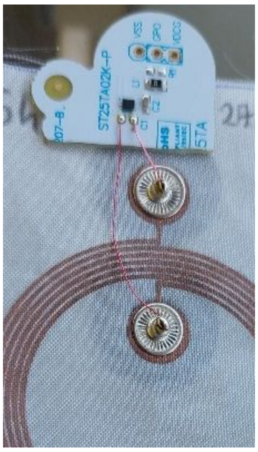

Figure S3. Metal buttons were attached to the textile antennas to enable connection to the NFC ST25TA02K-P chip.

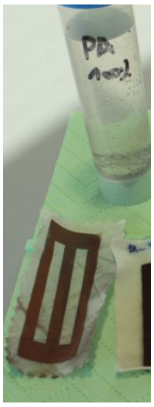

Figure S4. Photograph of the electroless copper deposition bath solution (45 mL) after extended deposition periods. The solution became transparent, indicating that all copper ions were reduced. An example of the Cu-coated woven polyamide fabric after removal from the bath solution is also shown.

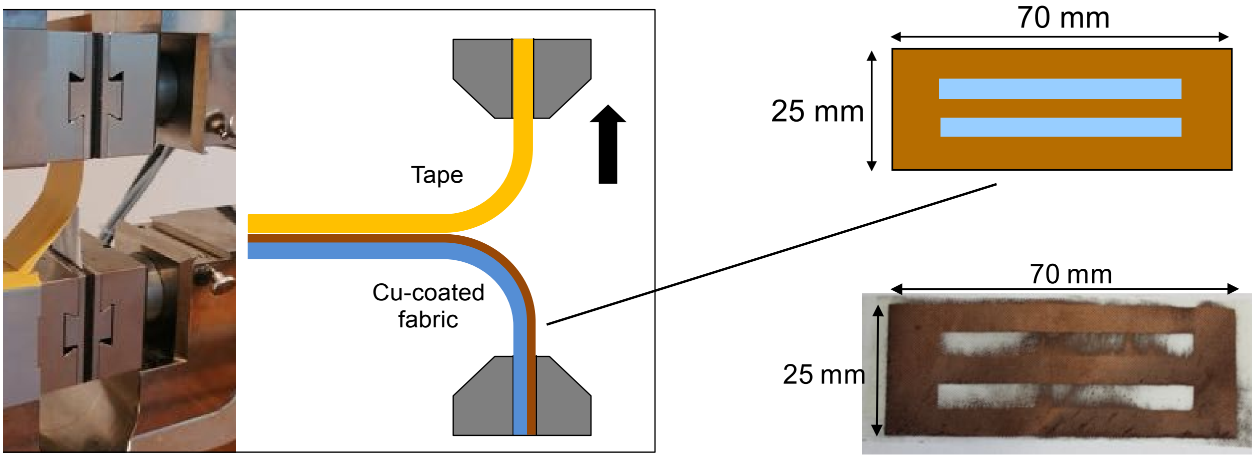

Figure S5. T-peel tests conducted on Cu-coated fabrics using a Zwick tensile strength machine. In the specimens, the blue layer represents siloxane-coated polyamide fabric, and the orange layer indicates the Cu coating.

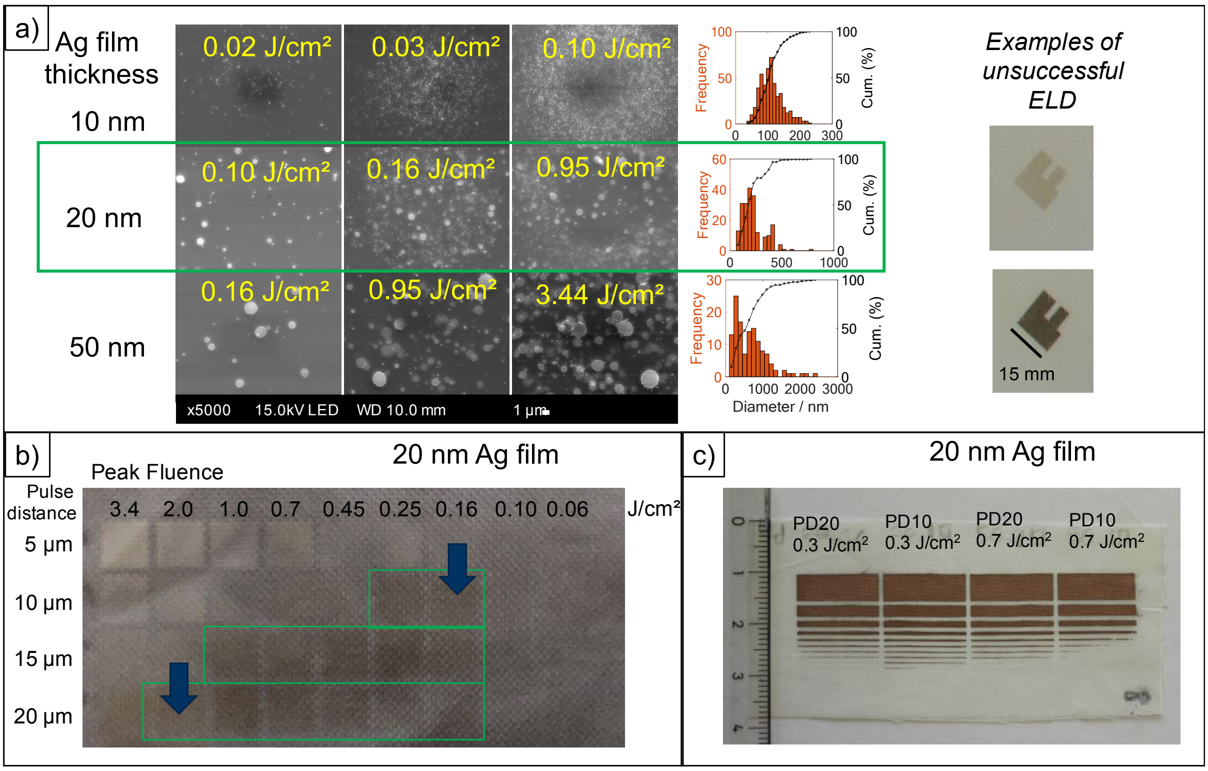

Figure S6. (a) SEM images of trasferred Ag particles on silicon wafer with 50 μm spacing using a Type 1 donor substrate with Ag film thicknesses of 10, 20 and 50 nm. We observed that the particle size distribution primarily depended on the Ag film thickness on the substrate donor, whereas the Ag particle yield and spatial distribution on the receiver were influenced by the peak fluence applied. The most suitable particle size distribution for subsequent electroless Cu deposition was obtained with a 20 nm Ag film, where 100% of the particles had a diameter below 1 μm, 80% below 200 nm, and 10% below 100 nm. When using 10 nm Ag film thicknesses, all particles had diameter below 200 nm with 50% smaller than 100 nm. In contrast, with a 50 nm Ag film, 50% of the particles exceeded 1 μm in diameter. SEM images suggest that particles close to or larger than 1 μm in diameter tend to resemble 2D circular film deposit rather than spherical morphology. This 3D morphology appears to be more favorable for electroless Cu deposition (ELD). However, when the Ag particles were too small (<100 nm), ELD was largely unsuccessful. Similarly, the particle morphology obtained with a 50 nm Ag film also led to poor deposition results. By unsuccessful, we refer to either a complete absence of Cu deposition or the formation of non-conductive Cu structures even at longer deposition times (more than 24 hours). Representative SEM images of these unsuccessful cases are shown.

Fluences between 0.09 and 0.3 J/cm^2 provided an appropriate yield and spatial distribution of particles. Although increasing the fluence led to a higher particle yield per cm^2, higher fluences also resulted in ring-shaped particle deposits, which were found to be less suitable for ELD. (b) Photograph of a fabric after LIFT using a Type 1 donor substrate with 20 nm Ag film thickness at varying pulse distances and peak fluences. Each condition was applied to an

irradiated area of 5 x 5 mm². Suitable conditions are highlighted in green. (c) Cu-coated fabric using Type 2 donor substrate under varying fluences of 0.3 and 0.7 J/cm² and pulse spacings 10 and 20 µm. These experiments served as the basis for selecting the subsequent LIFT conditions, which included various donor substrate types with 15-20 nm Ag film thicknesses, and fluences ranging from 0.09 to 0.3 J/cm².

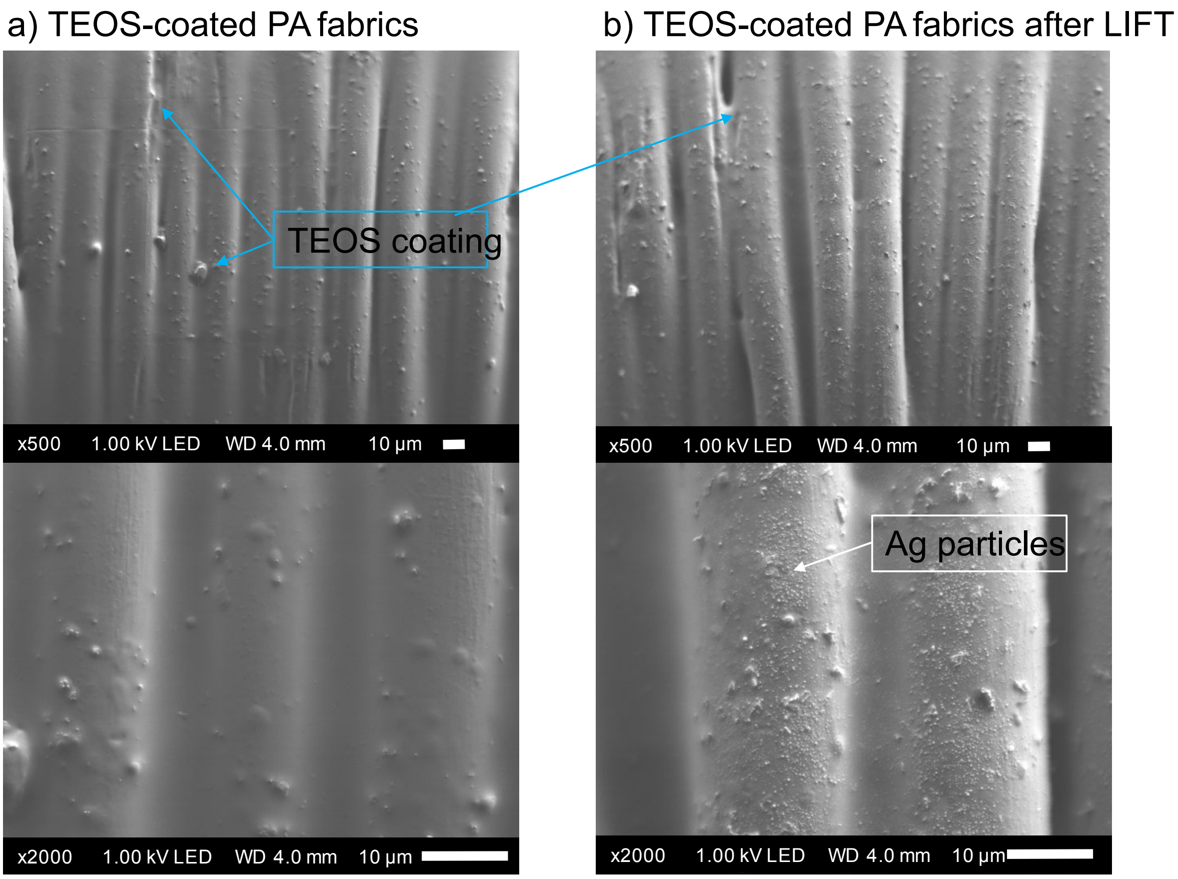

Figure S7. SEM images of the TEOS-coated PA fabrics before (a) and after LIFT (b). Visualization of the Ag particles becomes difficult on fabrics compared to glass substrates. A similar spatial distribution is expected for both fabrics and flat substrates depending on the donor substrate used. The fabrics consist of fibers with a diameter of 20 µm.

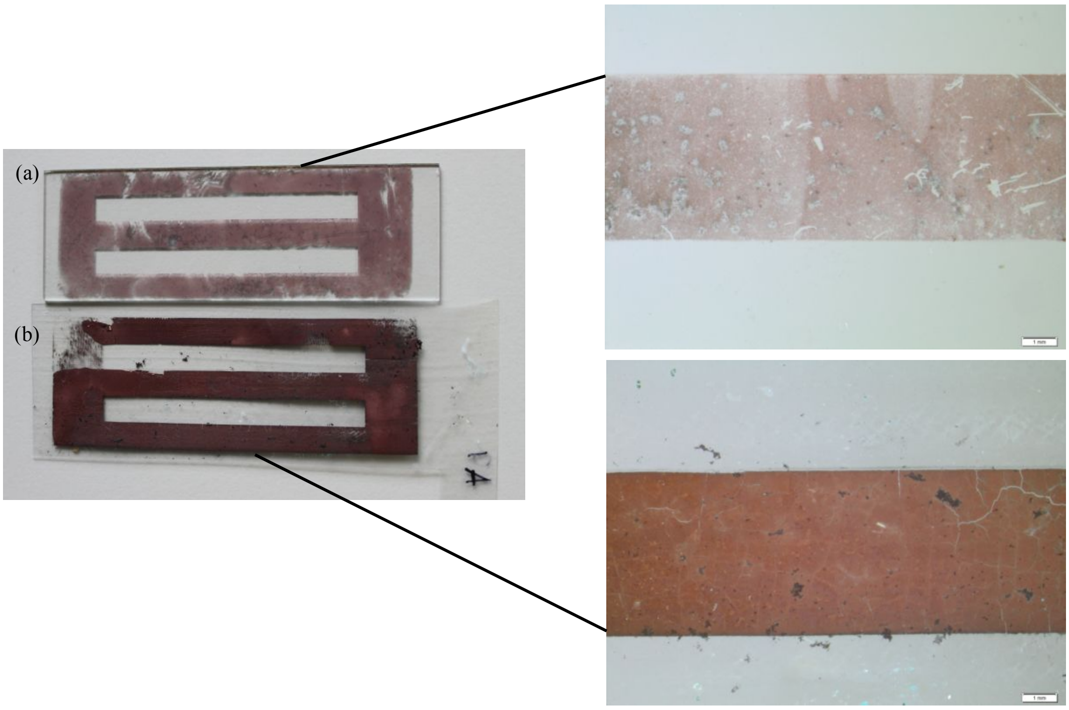

Figure S8. Photographs of the Cu coating on glass (a) and TEOS-coated PA film (b).

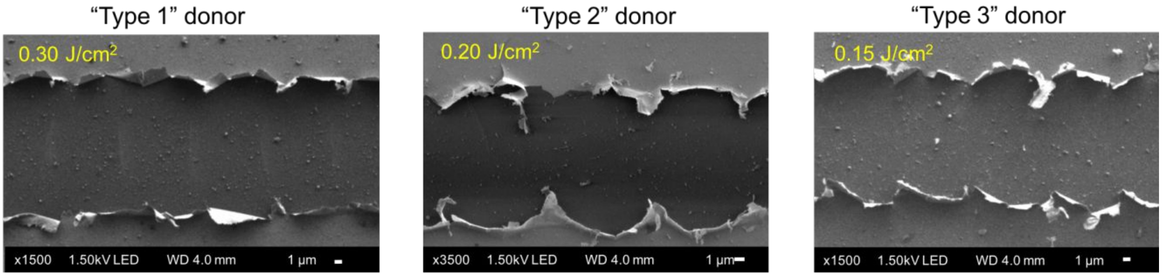

Figure S9. SEM images of the glass donors after the LIFT process using overlapping pulses with a pulse spacing of 10 µm.

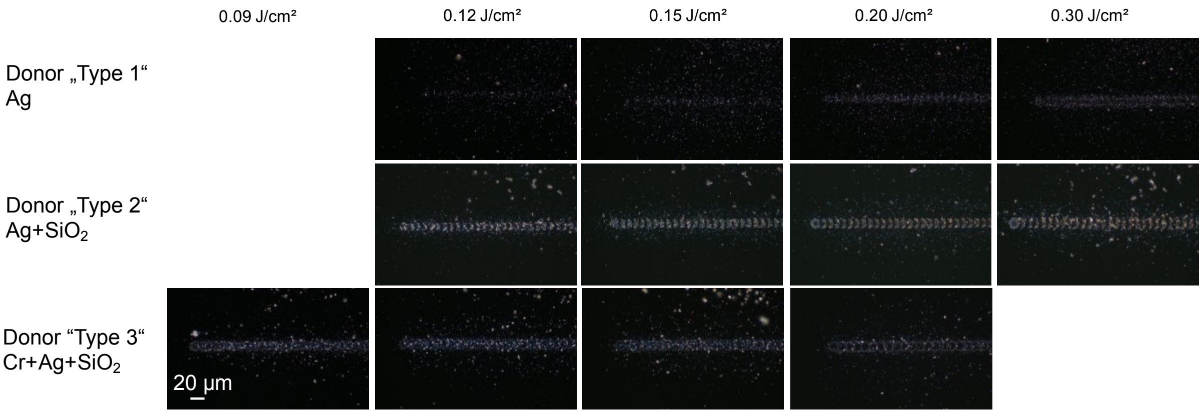

Figure S10. Dark-field optical images of the glass receiver after the LIFT process using overlapping pulses with a pulse

spacing of 10 μm. Optimal LIFT conditions for electroless Cu deposition correspond to those where transferred Ag is more uniformly distributed spatially on the receivers.

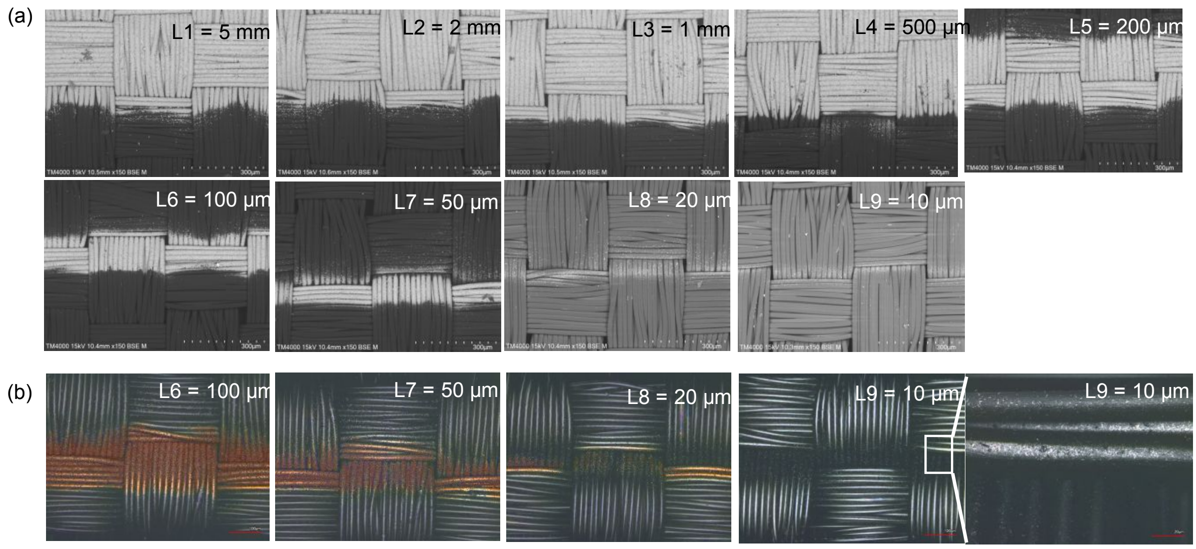

Figure S11. (a) SEM images x150 magnification of the Cu-coated fabrics obtained under optimal LIFT conditions with linewidths ranging from L1 = 5 mm to L9 = 10 μm. (b) Laser scanning confocal microscopic (LSM) images of the Cu-coated fabrics obtained with LIFT linewidths from L6=100 μm to L9=10 μm. The LSM images facilitate the identification of the Cu coating. For the L9 linewidth, a continuous Cu layer was not achieved.

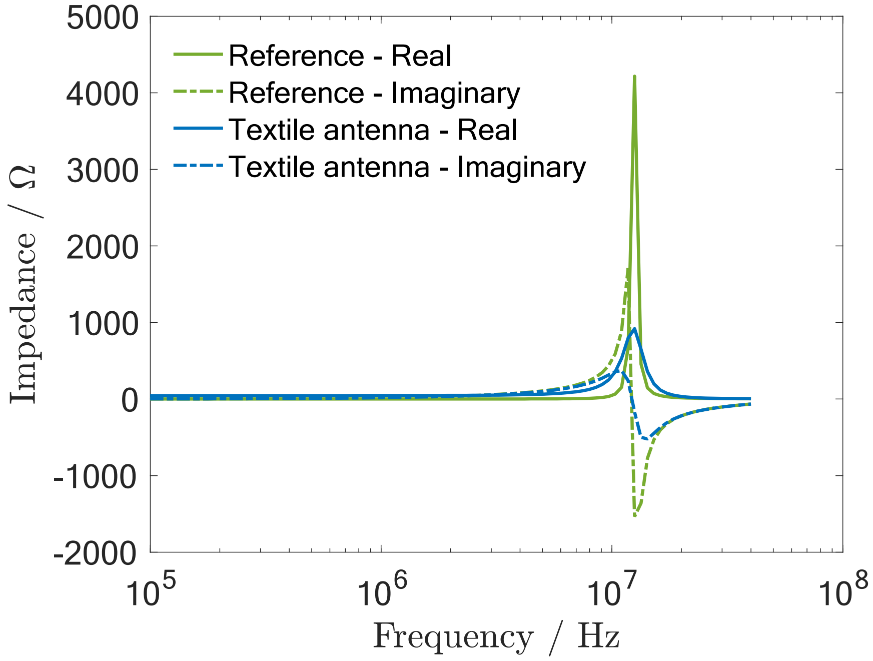

Figure S12. Impedance response of the FR4 rigid reference inductive antenna and the textile-based inductive antenna, each connected to an ST25TA02K-P chip to form a passive NFC tag IC operating at 13.56 MHz. A sharp peak around the operating frequency indicates that the antenna is well-tuned to the chip. Due to the difference in resistance values between the antennas, the reference antenna exhibited superior impedance characteristics at resonance compared to the textile-based antenna.

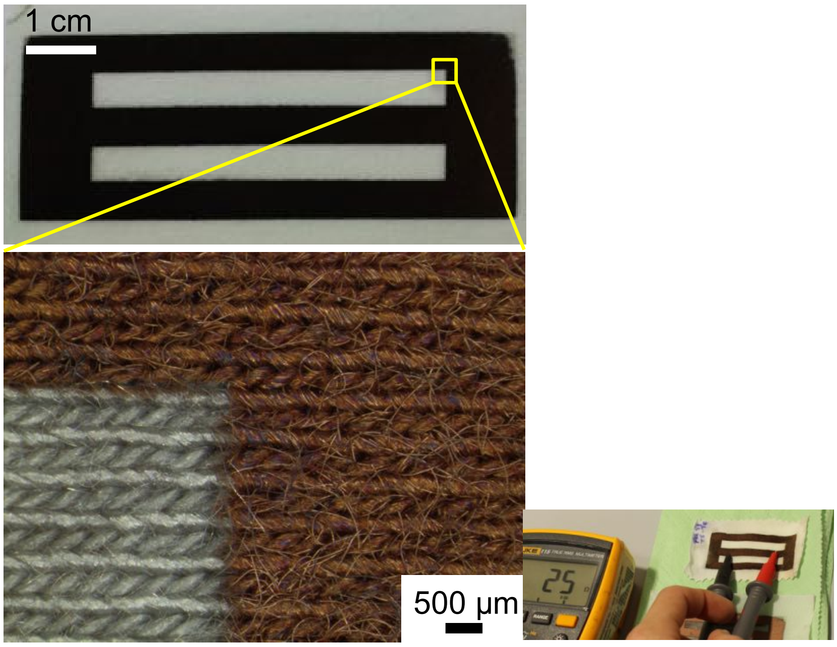

Figure S13. Cu-coated knitted fabrics (136 g/m^2, 93% polyamide and 7% elastane dtex 15/44/34 yarns, Wolford AG, Bregenz, Austria) prepared with the present method.

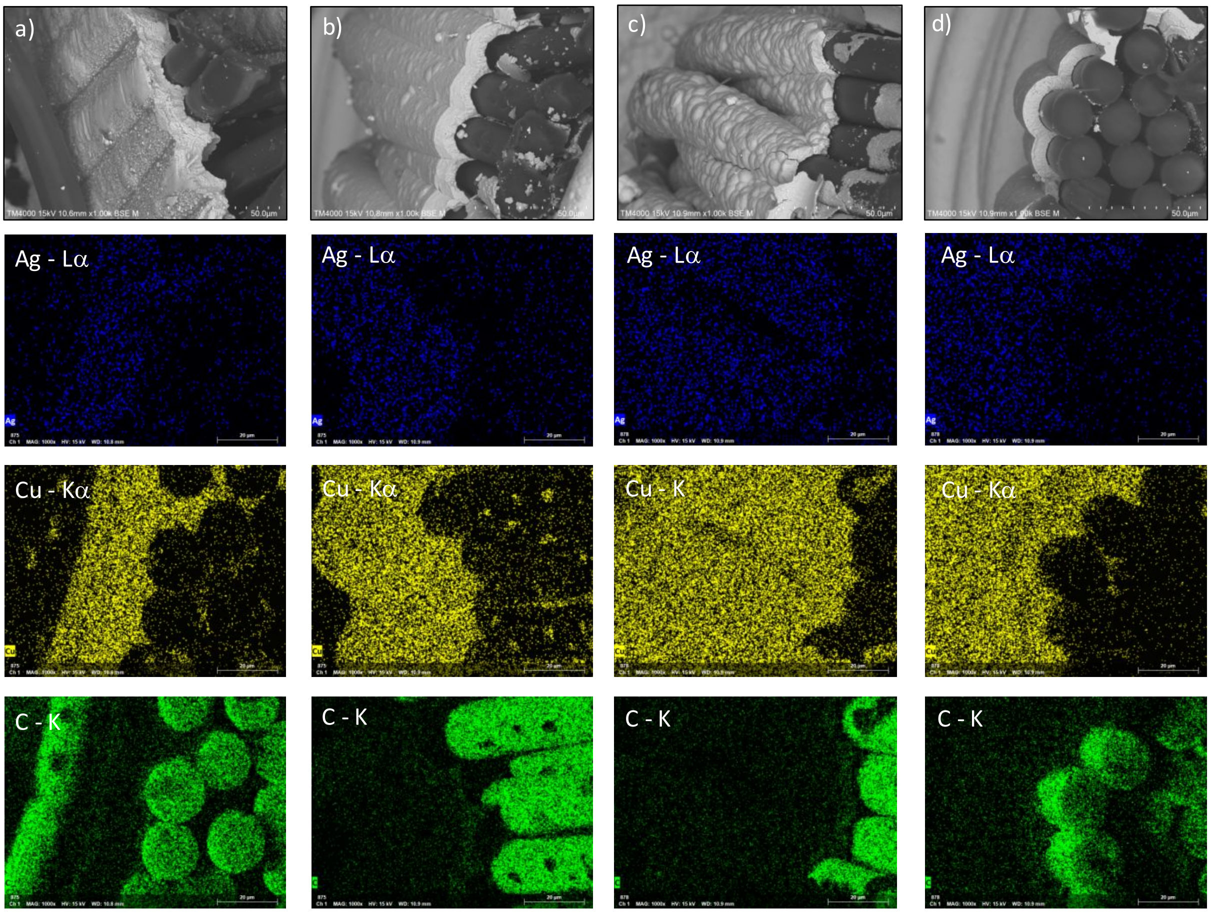

Figure S14. Cross-section SEM-EDX micrographs of the Cu-coated fabrics used for T-peel investigations: a) without siloxane intermediate coating, b) with TESPSA intermediate coating, c) with the combination TESPSA and TEOS precursors, and d) with TEOS intermediate coating.

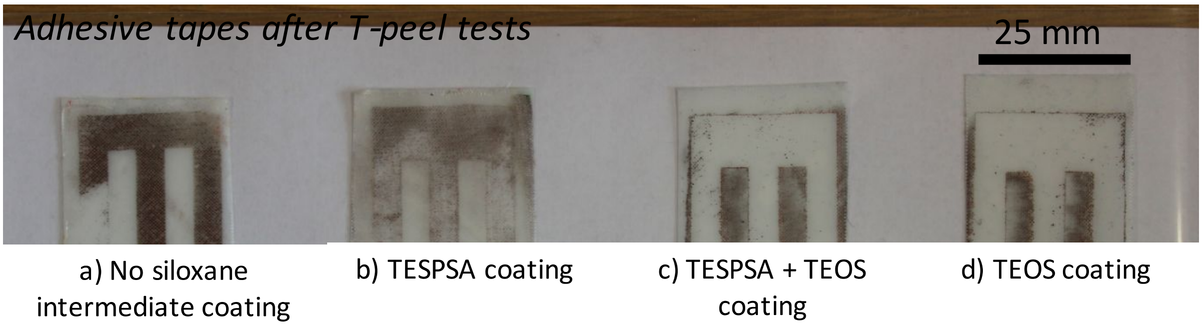

Figure S15. Pictures of the adhesive tapes after T-peel tests.

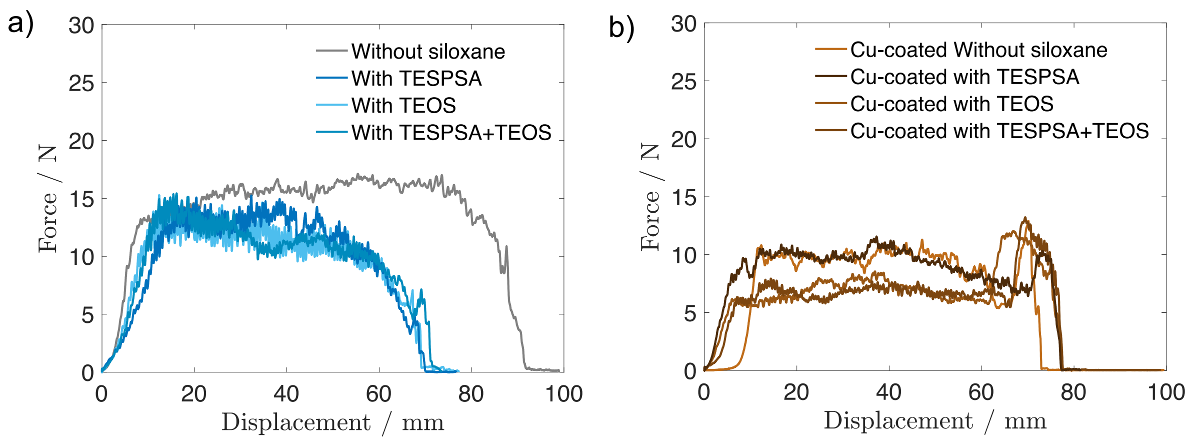

Figure S16. Peel force versus displacement curves from the T-peel tests for the fabrics: (a) before LIFT and electroless deposition (i.e., without Cu-coating), and (b) after electroless Cu deposition (i.e., with the Cu-coating).

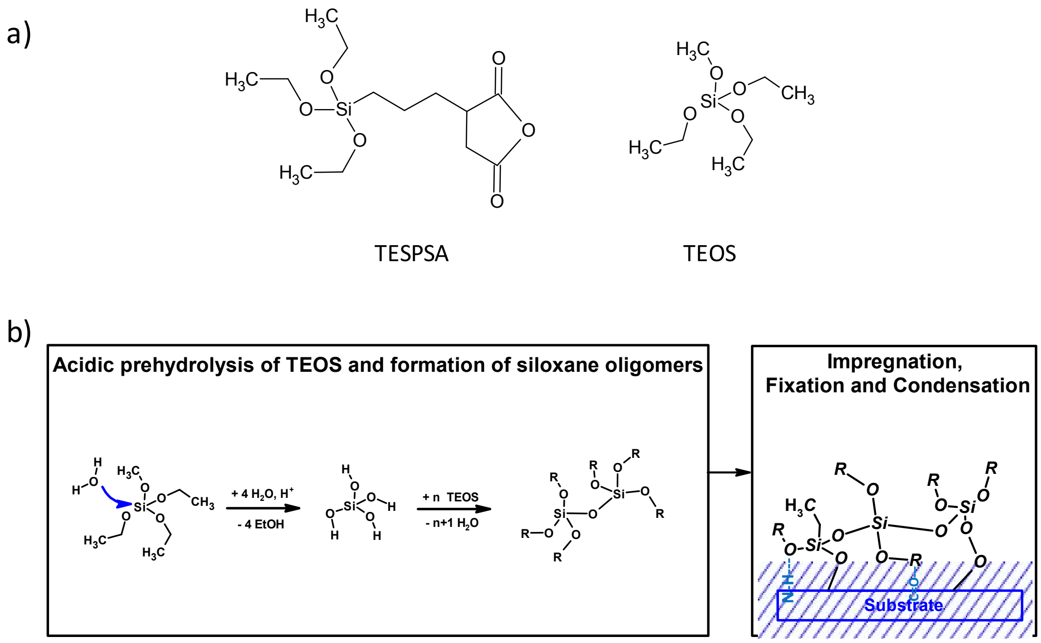

Figure S17. (a) Chemical structures of the TESPSA and TEOS precursors. (b) Reaction scheme of the siloxane coating with TEOS precursor applied to the polyamide fabric. The residuals R stand for either hydrogen groups or further monomers of the hydrolysed precursor.
